# Detection and Diagnosis of Breast Cancer Using Artificial Intelligence Based Assessment of Maximum Intensity Projection Dynamic Contrast-Enhanced Magnetic Resonance Images

**DOI:** 10.3390/diagnostics10050330

**Published:** 2020-05-20

**Authors:** Mio Adachi, Tomoyuki Fujioka, Mio Mori, Kazunori Kubota, Yuka Kikuchi, Wu Xiaotong, Jun Oyama, Koichiro Kimura, Goshi Oda, Tsuyoshi Nakagawa, Hiroyuki Uetake, Ukihide Tateishi

**Affiliations:** 1 Department of Surgery, Breast Surgery, Tokyo Medical and Dental University, Tokyo 113-8510, Japan; mioadachi1016@gmail.com (M.A.); oda.srg2@tmd.ac.jp (G.O.); nakagawa.srg2@tmd.ac.jp (T.N.); h-uetake.srg2@tmd.ac.jp (H.U.); 2 Department of Diagnostic Radiology, Tokyo Medical and Dental University, Tokyo 113-8510, Japan; m_mori_116@yahoo.co.jp (M.M.); kubotard@dokkyomed.ac.jp (K.K.); 11.ruby.89@gmail.com (Y.K.); commanderwxt@gmail.com (W.X.); ooymmrad@tmd.ac.jp (J.O.); kmrdrnm@tmd.ac.jp (K.K.); ttisdrnm@tmd.ac.jp (U.T.); 3 Department of Radiology, Dokkyo Medical University, Tochigi 321-0293, Japan

**Keywords:** breast imaging, magnetic resonance imaging, deep learning, convolutional neural network, object detection, artificial intelligence

## Abstract

We aimed to evaluate an artificial intelligence (AI) system that can detect and diagnose lesions of maximum intensity projection (MIP) in dynamic contrast-enhanced (DCE) breast magnetic resonance imaging (MRI). We retrospectively gathered MIPs of DCE breast MRI for training and validation data from 30 and 7 normal individuals, 49 and 20 benign cases, and 135 and 45 malignant cases, respectively. Breast lesions were indicated with a bounding box and labeled as benign or malignant by a radiologist, while the AI system was trained to detect and calculate possibilities of malignancy using RetinaNet. The AI system was analyzed using test sets of 13 normal, 20 benign, and 52 malignant cases. Four human readers also scored these test data with and without the assistance of the AI system for the possibility of a malignancy in each breast. Sensitivity, specificity, and area under the receiver operating characteristic curve (AUC) were 0.926, 0.828, and 0.925 for the AI system; 0.847, 0.841, and 0.884 for human readers without AI; and 0.889, 0.823, and 0.899 for human readers with AI using a cutoff value of 2%, respectively. The AI system showed better diagnostic performance compared to the human readers (*p* = 0.002), and because of the increased performance of human readers with the assistance of the AI system, the AUC of human readers was significantly higher with than without the AI system (*p* = 0.039). Our AI system showed a high performance ability in detecting and diagnosing lesions in MIPs of DCE breast MRI and increased the diagnostic performance of human readers.

## 1. Introduction

Breast cancer is the second most prominent cause of cancer-related death and the most common cancer affecting women [1]. The dynamic contrast-enhanced (DCE) magnetic resonance imaging (MRI) modality has the highest sensitivity, so it is widely used for breast cancer detection and diagnosis [2,3,4]. MRI screening is recommended annually for women at a high risk of breast cancer, in addition to annual mammography screening, particularly for women with a relative lifetime risk greater than 20% [5]. Recently, the abbreviated protocol for DCE breast MRI has been reported to provide comparable diagnostic performance to that of the conventional full protocol, increasing access to breast MRI and reducing the cost of existing MRI screening programs [6,7]. However, the lack of knowledgeable radiologists may limit the use of these MRI techniques and may result in unnecessary biopsies and follow-up assessments.

In recent years, artificial intelligence (AI), especially the deep learning (DL) method with convolutional neural networks (CNNs), has accomplished outstanding performances in medical breast imaging for pattern recognition, object detection, segmentation, and image synthesis [8,9,10,11,12]. Object detection—detecting instances of semantic objects of a certain class in digital images—is one of the most important computer technologies related to image processing. The technology of object detection using AI has been applied successfully to mammography image diagnosis [13,14].

Although some reports have built DL architecture to automatically extract features of images learned from MRI data and have evaluated an architecture to diagnose breast lesions [15,16,17,18,19], to our knowledge there is no report evaluating both AI and human interpretations of object detection on maximum intensity projections (MIPs) of DCE breast MRI. Because evidence of the usefulness of AI for object detection in breast MRI is inadequate, verification of its clinical utility is needed. Therefore, this study aimed to build an AI system to detect and diagnose lesions of MIPs of DCE breast MRI and evaluate their diagnostic performances and added value for human readers.

## 2. Materials and Methods

### 2.1. Patients

Our medical ethics committee approved this retrospective study and waived the requirement for written informed consent from patients. Inclusion criteria for enrolling patients in this study were (1) patients who underwent DCE breast MRI at our hospital between March 2014 and October 2018, and (2) patients who were diagnosed with benign or malignant lesions by pathology or a follow-up examination at more than one year. The following patients were excluded: (1) patients who were treated with breast surgery, hormonal therapy, chemotherapy, or radiation therapy; and (2) patients who were fewer than 20 years of age. A research assistant selected consecutive DCE breast MRI after reviewing the radiology report database and clinical records at our institute. The Medical Ethics Committee of Tokyo Medical and Dental University Hospital approved this retrospective study (approval ID: M2019-232, 13 September 2019) in accordance with the Helsinki Declaration and waived the requirement for written informed consent from the involved patients.

### 2.2. DCE Breast MRI Examinations

DCE breast MRI examinations were performed with a 3.0-T system (Signa HDxt, General Electric Medical Systems, Milwaukee, WI, USA) using a breast coil in the prone position. The early phase of a contrast-enhancement study within 1 and 2 min after intravenous bolus injection of Gd-DTPA (0.2 mL/kg) was acquired. Bilateral axial fat-suppressed T1-weighted sequence (TR/TE, 6.5/2.4; flip angle, 10°; 2 mm-thick section; 512 × 512 matrix; 360 mm field of view) was employed. Axial MIP images were reconstructed from the early phase of a DCE breast MRI

### 2.3. Data Set 

MIP breast MRIs were converted to jpeg format at a size of 512 × 512 pixels using the viewing software TFS-01 (Toshiba Medical Systems, Tochigi, Japan) for analysis. Table 1 shows the number, age, and image characteristics per patient as well as the number, image characteristics, image findings, and maximum diameter per breast. The training and validation data were distributed in a ratio of 3:1 (i.e., 214:72 cases), and the remaining 85 cases were used as test data. Cases were randomly distributed using random numbers. We used 30 normal, 49 benign, and 135 malignant cases as the training set; and 7 normal, 20 benign, and 45 malignant cases as the validation set. For the test phase, 13 normal, 20 benign, and 52 malignant cases were used.

Table 1 shows the histopathology of benign and malignant lesions in the training, validation, and test data.

Data augmentation (horizontal flip, brightness: dark, +20%; bright; −20%) were performed, and the images magnified six times were available for training and validation.

In this study, patients containing both benign and malignant lesions were defined as malignant cases, and individual breasts containing both benign and malignant lesions were also defined as malignant. In cases of multiple ipsilateral breast lesions, the largest lesion was recorded. Bilateral breast cancer was found in four training, five validation, and two test-phase patients.

**Figure 1:**
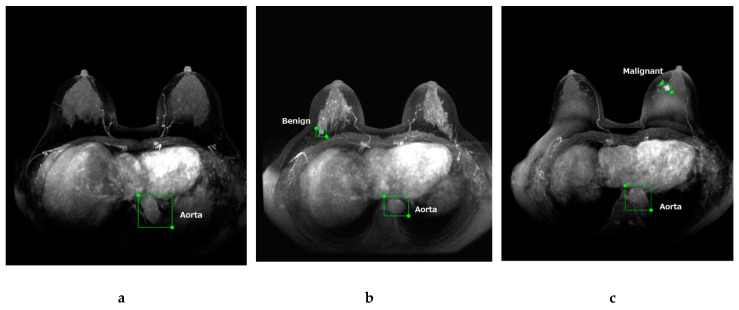
Maximum intensity projection of dynamic contrast-enhanced breast magnetic resonance images. Representative images of labeled normal (**a**), benign (**b**), and malignant (**c**) breast lesions.

Breast lesions were annotated with a rectangular region of interest (ROI) and labeled as “benign” or “malignant” by a breast radiologist (with 10 years of experience) after reviewing the radiology report database and clinical and pathological records at our institute. To label the normal images, the aorta in all images was also annotated with a rectangular ROI and labeled as “aorta”. In this study, we used the web-based open graphical image annotation tool labelme (https://github.com/wkentaro/labelme), and annotations to images were stored in COCO format. Representative images of labeled normal (Figure 1a), benign (Figure 1b), and malignant (Figure 1c) cases are presented in Figure 1.

### 2.4. AI System

Our study was performed on a Deep Station system (UEI, Tokyo, Japan) containing the graphics processing unit GeForce GTX 1080 (NVIDIA, Santa Clara, CA, USA), central processing unit Core i7-8700 (Intel, Santa Clara, CA, USA), and graphical user interface-based DL tool Deep Analyzer (GHELIA, Tokyo, Japan).

**Figure 2:**
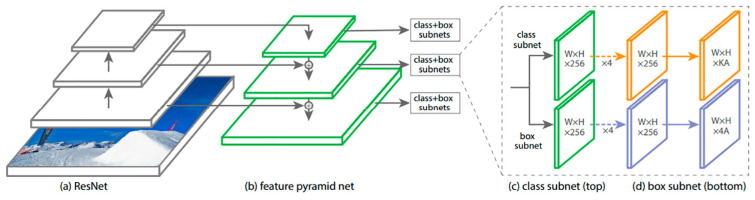
The RetinaNet architecture uses a Feature Pyramid Network [21] backbone on top of a feedforward ResNet architecture [22] (**a**) to generate a rich, multi-scale convolutional feature pyramid (**b**). To this backbone RetinaNet attaches two subnetworks: one for classifying anchor boxes (**c**) and one for regressing from anchor boxes to ground-truth object boxes (**d**). (Reprinted and adapted with permission from ICCV 2017 [20].)

DL architectures were constructed using RetinaNet [20]. RetinaNet is a one-stage object detector that was developed by Facebook AI Research in 2017. The authors of RetinaNet identified class imbalance as the most significant reason why the performance of a one-stage detector lags behind the performance of a two-stage detector. To improve performance, RetinaNet employs a simple and effective new loss function called Focal Loss, allowing more focus on difficult samples. RetinaNet uses a one-stage network architecture with Focal Loss to achieve high performance in terms of accuracy and running time [12,20]. Figure 2 shows the network architecture of RetinaNet.

**Figure 3:**
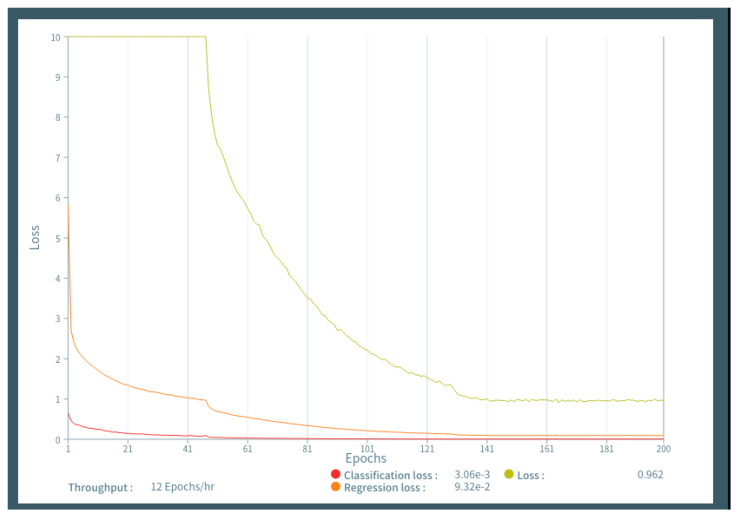
Figure 3 shows the learning curves generated by epochs and data loss, demonstrating that learning above 150 epochs resulted in low data loss.

The labeled images of DCE breast MRI were uploaded to the Deep Analyzer. An architecture without fine-tuning was conducted in supervised learning with 150 epochs using the training and validation image sets by the hold-out method. We evaluated the performance of the model with 50, 100, 150, and 200 epochs using training and validation data sets, and a 150-epoch parameter was selected with high accuracy and low data loss (Figure 3). In this study, there were two the holdout method was used rather than the n-fold cross-validation method. First, we used the commercially available graphical user interface-based DL tool Deep Analyzer. The model for deep learning was programmed to learn using the hold-out method. To use n-fold cross-validation and implement the consensus algorithm, we would need to ask the production company to reprogram it. Second, it takes more than half a day to perform just one set of training (150 epochs) on our computer. For example, 10 repetitions of five-fold cross validation will take more than a month of training time alone, even if it runs successfully. Because our study aimed to perform object detection of malignant lesions, our AI system was trained to detect and calculate the possibility of malignancy. We then examined the accuracy of the trained AI system to detect and diagnose breast lesions using the test image sets. With test data uploaded to the AI system, breast lesions were detected as rectangular ROIs, and the probability of malignancy (%) was calculated. 

### 2.5. Readout

Image findings of DCE breast MRI were retrospectively evaluated by four human readers (Reader 1 with 20 years, Reader 2 with 6, Reader 3 with 2, and Reader 4 with 1 year of experience in breast imaging) who were blinded to the clinical information and histopathological results of images. Sequential readings were performed per breast using test data sets of DCE breast MRI. First, the human readers detected lesions and recorded the possibility of malignancy (%) based on the Breast Imaging Reporting and Data System (BI-RADS) [23] per breast without the assistance of the AI system. Next, the human readers interpreted the image again with the assistance of the AI system and reported the possibility of malignancy (%) per breast. For scaling, BI-RADS 1 is normal with a malignancy rate of 0%; BI-RDAS 2 is benign with a malignancy rate of 0%; BI-RADS 3 is probably benign with a malignancy rate of ≤2%; BI-RADS 4a signifies a low suspicion of malignancy with a malignancy rate of >2% to ≤10%; BI-RADS 4b indicates moderate suspicion with a malignancy rate of >10% to ≤50%; BI-RADS 4c shows high suspicion with a malignancy rate of >50% to <95%; and BI-RADS 5 is highly suggestive of malignancy with a rate of ≥95%.

### 2.6. Statistical Analysis

A breast radiologist with 10 years of experience determined whether the AI system and human readers correctly detected and diagnosed malignant lesions based on medical records and pathological results.

All statistical analyses in this study were performed using the EZR software package version 1.31 (Saitama Medical Center, Jichi Medical University, Saitama, Japan) [24] and the Visualizing Categorical Data package version 1.4-4 with a graphical user interface for the R software package (version 3.5.1; R Development Core Team, Vienna, Austria). 

We performed a Mann–Whitney U-test to compare characteristics (age and maximum diameter at MRI) of patients with normal, benign, or malignant lesions. We analyzed sensitivity and specificity of detecting malignant lesions for the AI system and human readers (with or without the AI system) per breast. BI-RADS 4a (2%) was defined as the cutoff value. Moreover, receiver operating characteristic (ROC) analyses were performed to calculate the area under the curve (AUC) with 95% confidence intervals to determine their diagnostic performances using possibility of malignancy (%) and outcome (normal or benign vs. malignant) per breast. A *p*-value of <0.05 was considered to be statistically significant.

## 3. Results

Malignant lesions were larger than benign lesions according to MRI (*p* < 0.001), and patients with malignant lesions were also significantly older than other patients (*p* ≤ 0.041) in training, validation, and test sets. Although there was a significant difference in size between malignant masses for the test data and validation data (*p* = 0.023), in other cases there were no significant differences in age and size among the training, validation, and test data (Table 2). 

**Figure 4:**
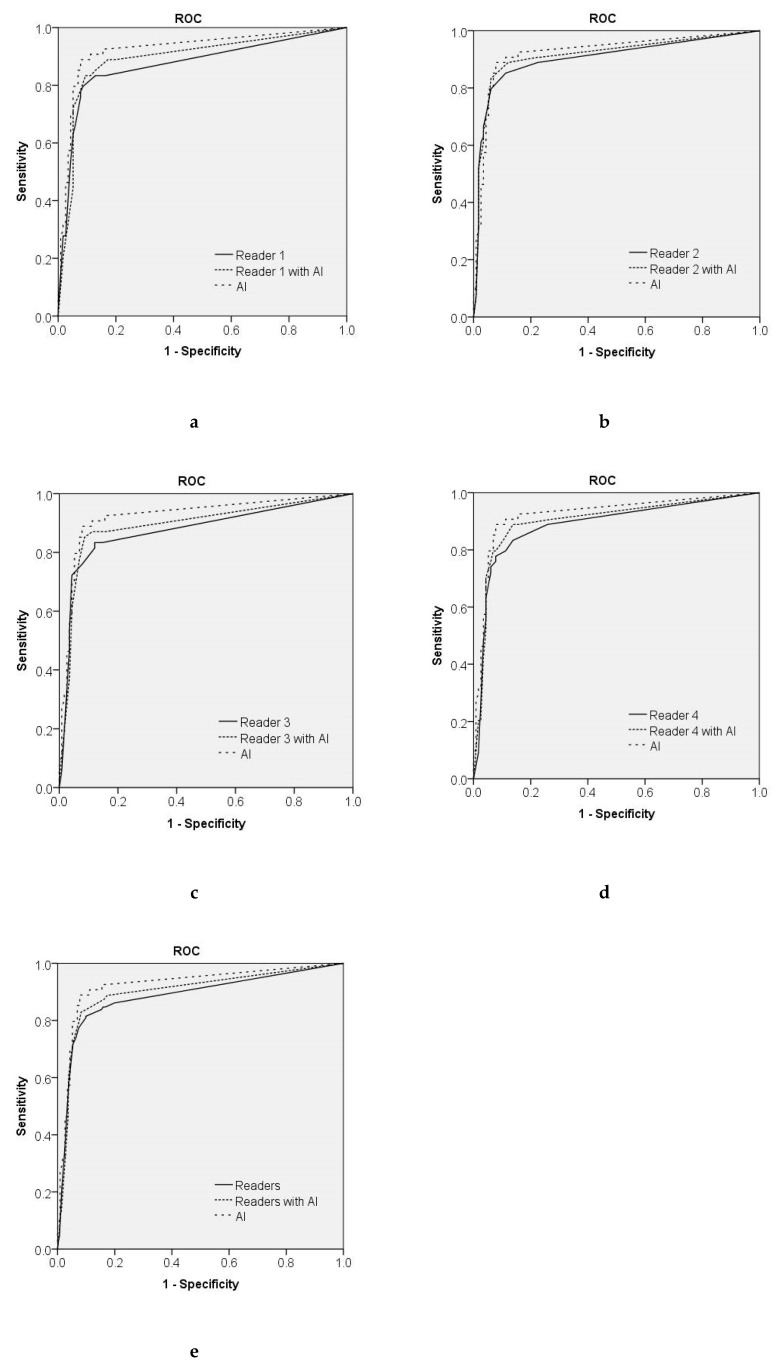
The ROC curve for the AI system compared with that of each human reader (**a**–**d**) and all four human readers (**e**). The AUCs of the human readers without the AI system were 0.872–0.904, and those of each human reader with the AI system were 0.893–0.915. The AUCs of the AI system, all four human readers without the AI system, and all four human readers with the AI system were 0.925, 0.884, and 0.899, respectively. The AI system showed better diagnostic performance compared with that of all four human readers without the AI system (*p* = 0.002), and the AUC of all four human readers using the AI system was significantly higher than that without the AI system (*p* = 0.039).

Table 3 and Figure 4 show the diagnostic performance of the AI system, human readers without the AI system, and human readers with the AI system.

Table 4 demonstrates a comparison of the diagnostic performance of the AI system, human readers without the AI system, and human readers with the AI system using ROC analyses.

**Figure 5:**
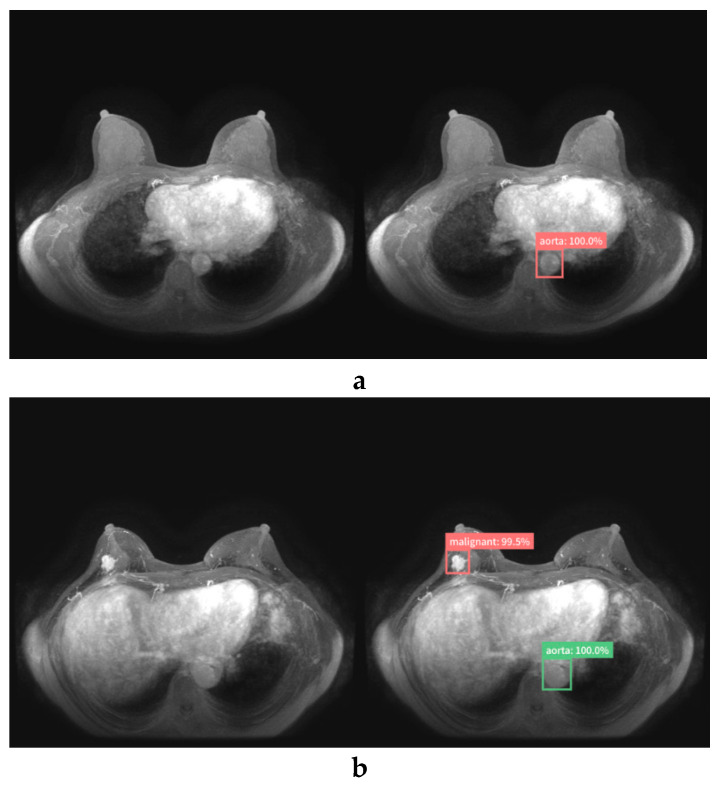

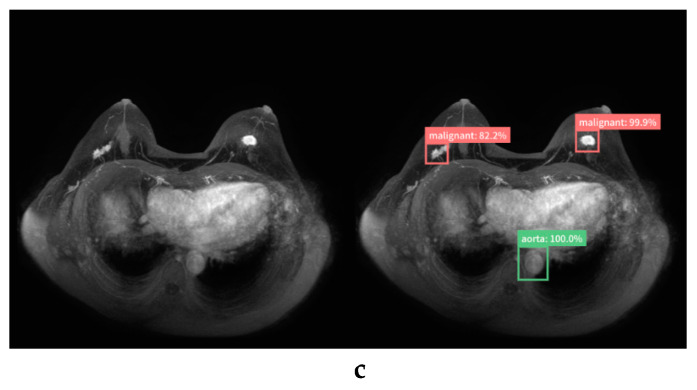
True-negative (**a**) and true-positive cases (**b**, **c**) diagnosed by the AI system (left, original image; right, image diagnosed by AI system). The AI system did not respond to normal breasts (**a**). The AI system correctly detected and diagnosed invasive ductal carcinoma (IDC) of the right breast (**b**) and invasive ductal carcinoma of bilateral breasts (**c**).

Sensitivity and specificity were 0.926 and 0.828 for the AI system, 0.847 and 0.841 for all four human readers without the AI system, and 0.889 and 0.823 for all four human readers with the AI system, respectively, using a cutoff value of 2%. The AUCs of the AI system, all four human readers without the AI system, and all four human readers with the AI system were 0.925, 0.884, and 0.899, respectively. The AUC of the AI system was higher than that of each human reader, and the AI system had a significantly higher AUC than any one of the human readers (*p* = 0.038). The AI system showed better diagnostic performance compared with all four human readers without the AI system (*p* = 0.002). Because of the increased performance of human readers with the assistance of the AI system, the AUC of all four human readers using the AI system was significantly higher than without the AI system (*p* = 0.039). Figure 5 shows the true-negative and true-positive cases.

Table 5 lists the MRI findings and pathological features of the cases that were diagnosed as false-positives (i.e., benign lesions judged to be 50% malignant by the AI system) and false negatives (i.e., malignant lesions judged to be 2% benign by the AI system). 

**Figure 6:**
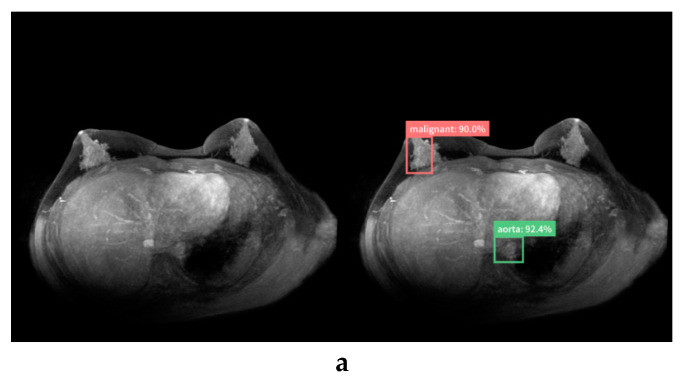

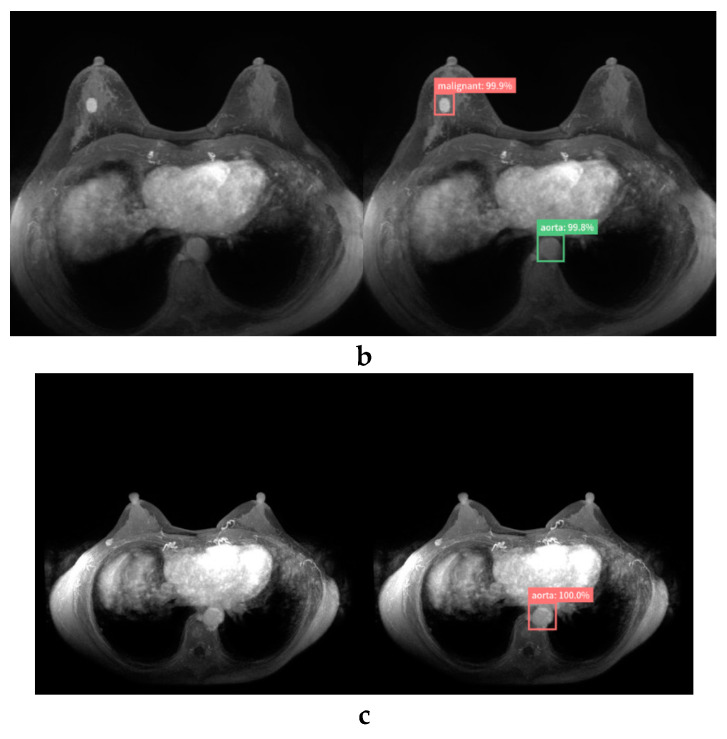
False-positive case (**a**,**b**) and false-negative case (**c**) diagnosed by the AI system (left, original image; right, image diagnosed by the AI system). The AI system mistakenly detected normal breasts (**a**) and fibroadenoma (**b**) and diagnosed them as malignant. The AI system failed to detect invasive ductal carcinoma near the right axilla. This may have been mistaken for an axillary lymph node (**c**).

Figure 6 shows the false-positive and false-negative cases. 

In the case of seven false-positives, five lesions were masses (mean size = 15.6 mm; range, 9–27 mm), three were diagnosed as fibroadenoma or intraductal papilloma (IDP), and the remaining two were clinically diagnosed as benign. Almost half of the human readers had false-positives, as did the AI system. Two lesions were normal breast tissue without abnormal findings from MRI. Most readers, unlike the AI, were able to correctly diagnose normal breasts. In the case of four false negatives, three lesions were masses (mean size, 9.3 mm; range, 7–11 mm) diagnosed as invasive ductal carcinoma (IDC), and one lesion was non-mass (size: 17 mm) diagnosed as a non-invasive ductal carcinoma. Two masses were diagnosed as malignant by half of the readers. The remaining two were misdiagnosed as normal by all human readers and the AI system.

## 4. Discussion

Recently, a DL method has demonstrated outstanding performance compared with that of conventional computer methods in medical imaging. DL is an area of AI in which computers are not explicitly programmed but can analyze relationships between existing data and perform tasks based on these new data [25]. 

Our study is the first report to focus on building an AI system based on RetinaNet that detects and diagnoses lesions of MIPs of DCE breast MRI and compares the diagnostic performance of the AI to that of human readers. In addition, we examined the added value of the AI system in support of human readers. 

Our AI system had a statistically higher diagnostic performance (AUC, 0.925) than that of experienced human readers (AUC, 0.884; *p* = 0.002), and the support of the AI systems significantly improved the diagnostic performance of human readers (AUC, 0.899; *p* = 0.039).

Because MRI screening is recommended annually for high-risk patients with breast cancer [5], and the abbreviated MRI protocol enables cost-effective and efficient testing, the amount of breast MRI is expected to increase in the future. To reduce the resulting cost of interpretation and the burden on radiologists, it will be important to develop and clinically apply an AI system that can efficiently detect and diagnose breast lesions on MIPs of DCE breast MRI. Our pilot evaluation herein has provided the necessary groundwork for clinical trials to improve the accuracy and efficiency of MRI breast cancer screening with an AI system. The proportion of malignant cases in this study was relatively high. Whether our AI system is useful for populations with few malignant cases, such as when using screening tests, will be validated in future research.

In this study, we used RetinaNet, a state-of-the-art one-stage object detector that has been successfully applied to medical images such as mammography, computed tomography, and x-ray examinations [12,26]. We have shown that it is useful for detecting and diagnosing breast lesions on MIPs of DCE breast MRI.

In the present study, we tested the diagnostic performance of human readers with various years of experience in breast MRI. Reader 4 with only one year’s experience performed similar to the experienced readers. This may be because interpretation of MIP images is simpler than that of full-study DCE-MRI images. Referencing images annotated with lesion locations and probabilities of malignancy (%) according to the AI system, the AUCs of each human reader were increased. This result suggests that our AI system may be useful not only for beginners but also for experts. Similar to our results, other studies have reported the utility of DL for breast MRI [15,16,17,18]. Hernet et al. developed a supervised-attention model with DL based on ResNet-50 that was trained to detect and characterize lesions using a single two-dimensional T1-weighted fat-suppressed contrast-enhanced MRIs of 335 cases. The performance of the model was evaluated on an independent test set of 168 MRIs, and an AUC of 0.893 was obtained to distinguish benign and malignant lesions [17]. 

In contrast with our results, Truhn et al. compared human performances with those of radiomic algorithms and DL in breast lesion recognition using multiparametric MRI, and showed that interpretation by a radiologist was better (AUC, 0.98) than that by a CNN (AUC, 0.88) [19]. Because our study using only one MIP image was simpler than their study using multiparametric MRI data, our AI system possibly had a better diagnostic performance than human readers did. Thus, evidence of the usefulness of DL in breast MRI is still inadequate and premature, and further verification is needed.

We also examined the MRI findings, results of human readers, and pathological features of breast lesions that were misdiagnosed by the AI system. There were seven false-positive and four false-negative cases. There were some cases that human readers could correctly diagnose but the AI system could not. The AI system mistakenly detected two normal breasts and misdiagnosed them as malignant. Because there are many variations in breast tissue (e.g., breast density and background parenchymal enhancement), an untrained pattern of normal breasts may have been detected as lesions. In addition, the AI system was unable to detect invasive ductal carcinoma near the axilla, and the AI system may have mistakenly identified these as normal axillary lymph nodes. Training with a wider variety of data may solve these problems, and it may be possible to build a more powerful AI system in this manner. Furthermore, if human readers comprehend the advantages and disadvantages of an AI system based on the results of this study and use the AI system effectively, their diagnostic performance with the AI system may be further improved.

There are several limitations to this study. First, some lesions were diagnosed by clinical follow-ups rather than histological diagnosis. Second, we performed this study using images that were converted to a size of 512 × 512 pixels in jpeg format. This image processing can result in information loss and thereby influence the performance of the AI system. Third, this study uses DCE fat-suppressed T1-weighted axial imaging. If fat suppression is poor, areas of insufficient fat suppression can be mistaken for contrast lesions, which can influence the performance of the AI system. In summary, we built an AI system based on the hold-out method, which represents the simplest kind of cross validation method and exploits just one data split. The performance of the model depends on how the data are split, which could lead to errors in its evaluation. An N-fold cross-validation method is potentially more effective for deep learning based on multiple training-validation splits and should provide a better indication of how well a model would perform given new previously unseen data [27,28]. An N-fold approach is currently beyond the capability of our resources, but future research will investigate whether an n-fold cross-validation method can be used to construct a highly accurate and stable AI system.

In conclusion, our AI system showed high performance ability in detecting and diagnosing lesions of MIPs of DCE breast MRI, and increased the diagnostic performances of human readers. Therefore, this AI system may be useful for radiologists in object detection in breast MRI.

## Figures and Tables

**Table 1:** Histopathology of benign and malignant lesions.

Training Data		Validation Data		Test Data	
Benign (*n* = 49)	Malignant (*n* = 135)	Benign (*n* = 20)	Malignant (*n* = 45)	Benign (*n* = 20)	Malignant (*n* = 52)
Fibroadenoma 8	Ductal Carcinoma In Situ 22	Fibroadenoma 4	Ductal Carcinoma In Situ 2	Fibroadenoma 4	Ductal Carcinoma In Situ 3
Papilloma 9	Invasive Ductal Carcinoma 91	Papilloma 3	Invasive Ductal Cancer 29	Papilloma 3	Invasive Ductal Carcinoma 38
Mastopathy 4	Mucinous Carcinoma 3	Mastopathy 3	Mucinous Carcinoma 2	Mastopathy 3	Mucinous Carcinoma 1
Benign Phyllodes Tumor 2	Invasive Lobular Carcinoma 7	Non-Specific Benign Lesion 2	Invasive Lobular Carcinoma 5	Non-Specific Benign Lesion 2	Invasive Lobular Carcinoma 1
Non-Specific Benign Lesion 4	Apocrine Carcinoma 2	Not Known * 8	Apocrine Carcinoma 1	Not Known * 8	Apocrine Carcinoma 1
Not Known * 22	Malignant Phyllodes Tumor 1		Malignant Phyllodes Tumor 1		Malignant Phyllodes Tumor 2
	Unclassifiable 9		Unclassifiable 5		Unclassifiable 6

*: Clinically diagnosed by observation, **: Diagnosed by fine needle aspiration.

**Table 2:** Characteristics of patients and images.

		Normal	Benign	Malignant
Training Data	Patients (n)	30	49	135
	Age (years)	38–72	38–74	26–86
	Range, Mean ± SD	52.9 ± 11.1	46.3 ± 11.0	58.6 ±12.8
	Breasts (n)	201 ^a^	88	139
	Mass/Non-mass (n)		77/11	114/25
	Size at MRI (mm)		3–105	6–95
	Range, Mean ± SD		14.9 ± 15.0	23.6 ± 16.9
Validation Data	Test Data (n)	7	20	45
	Age (years)	40–54	28–79	26–78
	Range, Mean ± SD	46.0 ± 5.6	50.0 ± 12.5	55.6 ± 13.1
	Breasts (n)	64 ^a^	30	50
	Mass/Non-mass (n)		22/8	38/12
	Size at MRI (mm)		3–62	6–123
	Range, Mean ± SD		17.5 ± 15.2	35.0 ± 26.8
Test Data	Patients (n)	13	20	52
	Age (years)	21–77	20–79	30–85
	Range, Mean ± SD	47.2 ± 12.6	47.2 ± 11.1	58.3 ±13.6
	Breasts (n)	92 ^a^	24	54
	Mass/Non-mass (n)		19/5	47/7
	Size at MRI (mm)		5–33	7–106
	Range, Mean ± SD		13.6 ± 8.8	25.6 ± 22.0

SD: Standard deviation. ^a^: “Normal breasts” is the total number of bilateral breasts of normal patients and contralateral normal breasts of benign and malignant patients.

**Table 3:** Diagnostic performance of human readers and the AI system.

	Sensitivity	Specificity	AUC (95% CI)
AI	0.926	0.828	0.925 (0.878–0.971)
Reader 1	0.833	0.836	0.872 (0.811–0.933)
Reader 1 with AI system	0.889	0.802	0.893 (0.837–0.948)
Reader 2	0.889	0.776	0.904 (0.849–0.959)
Reader 2 with AI system	0.907	0.759	0.915 (0.863–0.967)
Reader 3	0.833	0.853	0.876 (0.816–0.936)
Reader 3 with AI system	0.874	0.844	0.893 (0.847–0.956)
Reader 4	0.862	0.833	0.887 (0.829–0.945)
Reader 4 with AI system	0.889	0.845	0.902 (0.847–0.956)
All Human Readers	0.847	0.841	0.884 (0.854–0.920)
All Human Readers with AI system	0.889	0.823	0.899 (0.872–0.929)

Cutoff value was defined as 2%. AI: artificial intelligence; AUC: area under the receiver operating characteristic curve; CI: confidence interval.

**Table 4:** Statistical Analysis of the AUCs of Human Readers and AI.

Reader without AI System	*p*-Value (vs. AI System)	*p*-Value (vs. Reader with AI System)
Reader 1	0.038	0.203
Reader 2	0.414	0.200
Reader 3	0.076	0.393
Reader 4	0.143	0.203
Readers	0.002	0.039

The *p*-value was calculated by comparing AUCs. AI: artificial intelligence; AUC: area under the receiver operating characteristic curve.

**Table 5:** Pathological features of false-positive and false-negative cases diagnosed by AI.

No	Size (mm)/Mass or Non-Mass	Possibility of Malignancy (%)					Pathology
		AI	Reader 1	Reader 2	Reader 3	Reader 4	
False Positive							
1	27 mm/mass	66.2	10	0	0	30	Observation
2	No findings	57.9	0	10	0	0	Normal
3	No findings	90.0	0	0	1	0	Normal
4	19 mm/mass	99.9	80	60	10	90	Fibroadenoma
5	9 mm/mass	83.5	2	20	0	1	IDP
6	9 mm/mass	97.7	60	20	0	10	Observation
7	14 mm/mass	99.9	95	40	5	95	Fibroadenoma
False Negative							
1	10 mm/mass	0	0	50	0	90	IDC
2	11 mm/mass	0	0	0	0	0	IDC
3	17 m/non-mass	0	0	0	0	0	DCIS
4	7 mm/mass	0	50	10	1	20	IDC

AI: artificial intelligence; DCIS: ductal carcinoma in situ; IDC: invasive ductal carcinoma; IDP: intraductal papilloma.
